# Area Health Education Center (AHEC) programs for rural and underrepresented minority students in the Alabama Black Belt

**DOI:** 10.1186/s13690-017-0200-1

**Published:** 2017-07-24

**Authors:** Ashruta Patel, Regina J. Knox, Alicia Logan, Katie Summerville

**Affiliations:** 1 Philadelphia College of Osteopathic Medicine—Georgia Campus, 625 Old Peachtree Road NW, Suwanee, GA 30024 USA; 2 West Central Alabama—Area Health Education Center, 800 Hall St., Suite C, Greensboro, AL 36744 USA

**Keywords:** Area Health Education Center, West Central Alabama, Alabama Black Belt, Rural, Minority, Underserved

## Abstract

**Background:**

This paper evaluated the implementation West Central Alabama Area Health Education Center programs for high school students in grades 9–12 through participant-reported evaluations and feedback during the  September 1st, 2013 to August 31st, 2014 fiscal year. The programs targeted racial/ethnic minorities and/or rural individuals interested in pursuing a career as a healthcare provider in medically underserved counties of Alabama.

**Methods:**

Students participated in enrichment activities related to prospective health careers that included: successful college preparedness, knowledge about health careers, and the types of primary care health professions that are needed in underserved Alabama communities. The curriculum studied 593 (ACT preparation: *n* = 172, AHEC 101: *n* = 56, FAFSA: *n* = 109, Health Career Exploration: *n* = 159, College Career Readiness: *n* = 67, Dixie Scholars NERD: *n* = 30) baseline measures for the programs to evaluate effectiveness when rated by participants both quantitatively and qualitatively.

**Results:**

Interactive activities with video incorporation, hands-on experiences, and group discussions paired with student motivation and interest in specific health career-related activities provided the highest program ratings.

**Conclusions:**

It is important to use a variety of successful program strategies when forming healthcare workforce development interventions. Student evaluations can help adapt methods for future program implementation to ultimately achieve strategies for health professional recruitment, training, and retention in areas that lack access to quality healthcare.

## Background

After the abolishment of slavery, the term “Black Belt” was used to describe regions with high percentages of African Americans. The term originates from the dark soil located in regions of the Southern United States and is generally used to describe areas in the Deep South [1]. The Alabama Black Belt consists of communities that often have higher rates of poverty, low literacy levels, and poorer health outcomes compared to the rest of the country. This historically has been attributed to a variety of factors, including lack of access to adequate healthcare services, higher unemployment rates, transportation and limited access to quality health care, which impact overall healthcare outcomes across all demographics [1–3]. People who live in the Alabama Black Belt region often have co-morbidities and higher mortality rates when compared to the national average, and due to this, Alabama has some of the poorest health outcomes in the nation [2].

To address Alabama’s low overall health ranking in the country, Area Health Education Centers (AHECs) throughout the state are researching ways to expand the health care workforce that are most effective for the communities and populations they serve by increasing diversity in the healthcare workforce, reducing health disparities, and improving health outcomes with the ultimate goal to increase the quality of healthcare in rural and underserved communities [4]. The Alabama Statewide AHEC program is comprised of five community-based centers that serve all 67 Alabama counties.

AHECs were created in 1972 by Congress to enhance access to primary care by increasing the number of health providers in rural and underserved communities across the United States [5, 6]. Studies have found various factors influencing AHECs’ ability to provide appropriate resources to younger students, including sustained focus on recruiting adolescents into health professions, establishing links with institutions and organizations, and leveraging resources from partnerships [7]. Many AHECs across the country are successful at recruiting and retaining health professionals in rural and underserved communities by creating rural primary care residencies and collaborating with academic institutions to incorporate community-based rotations for health profession students that have performance-based approaches and interprofessional learning strategies [8, 9]. Some AHEC initiatives combine student experiences with minority recruitment efforts through the following methods: housing and travel assistance; educational support through curriculum development and rotation coordination; exposure to health-related barriers for rural and underserved communities, notably within minority populations; logistical assistance by way of telecommunication and seminars; translation help; and finally, provider support through fairs and conferences for continuing education needs [10].

The current health care system presents additional issues for vulnerable communities where resources and access to quality health care is compromised. Minority participation in tailored programs can help improve educational training experiences and increase interests in high demand health professions that are essential to improve current health outcomes. Receiving training in environments with social and cultural aspects of rural residence has the potential to create health professionals who choose to stay longer in rural practice, while at the same time feel more prepared to serve similar communities [10]. The Health Resources and Services Administration (HRSA) financially supports national AHECs to help recruit, train, and retain health professionals for medically-underserved communities by providing academic and clinical resources within the community to foster appropriate health needs. In addition, studies suggest that collaboration with community health centers can help balance the AHEC education-service mission and facilitate academic needs with workforce priorities [11].

This study explores the usefulness of various West Central Alabama AHEC (WCAAHEC) programs. Objectives of this study include: 1) Evaluating the effectiveness of WCAAHEC health professional enrichment programs for rural and underrepresented minority students; 2) Establishing a pathway for future WCCAHEC program development for cohorts of rural and underserved minority students; 3) Assessing the importance of health professional program implementation to effectively care for patient populations residing in the Alabama Black Belt.

## Methods

The baseline study was implemented from September 1st, 2013 to August 31st, 2014 for high school students that participated in WCAAHEC’s activities for future intervention development to recruit minority health professional students for employment in the Alabama Black Belt. The inclusion criteria for each participant in the program included the following: participants available to partake in the various enrichment programs over the required time period; participants from a medically underserved county in the 13-county service area; and participants interested in pursuing a health career in a rural or undeserved community. The program year recruited participants from four Alabama counties: Tuscaloosa, Greene, Dallas, and Wilcox. Participants self-reported minority and disadvantaged student status and all unknown responses were removed from the sample population.

### Program sessions and activities

#### ACT preparation program

Students were provided with a four-part review of the American College Testing (ACT) college readiness assessment, sample questions, and effective test-taking strategies information. Students had the opportunity to win ACT prep books and additional incentives such as water bottles, gift cards, USB thumb drives, bags, and t-shirts in exchange for participation.

#### FAFSA program

Students were provided with hands-on assistance in completing the Free Application for Federal Student Aid (FAFSA). Prior to the workshop, students received paper worksheets to take home to complete with their parents. During the workshop, students brought their completed worksheets and had the option to submit their FAFSA application online. Students learned about the different types of financial aid available to them and the next steps to take after completing the application.

#### AHEC 101

This presentation provided an overview of AHEC at the national, state, and community level. Participants learned about the three core programs of AHEC which include: community-based student education; health careers preparation and promotion; and lastly, professional education and support. The presentation also provided students with information on scholarship programs and health career focused programs offered by academic institutions.

#### The Dixie AHEC Scholars Program (DASP)

This was a school-based enrichment program designed to expose high school students from rural, disadvantaged, or underserved areas in West Alabama to various health careers. Students were provided accurate and relevant health career information through an enrichment series consisting of eight sessions featuring various healthcare professions. Sessions also included college readiness and preparatory skills. Each DASP session was designed to be implemented during a typical class period, approximately 45–50 min. The session format included a video presentation on a topic followed by a short PowerPoint presentation on the session’s subject matter, and a health career activity to reinforce the content covered. DASP included the following information sessions, which are outlined in detail below: pharmacy, dentistry, public health, nursing, medicine, nutrition/geriatric sensitivity, college preparation/financial aid, and a Survey Monkey pre-test and post-test assessment.

#### Pharmacy session

Students were introduced to pharmaceutical careers through games and simulations. A video about pharmacists and what the occupation entails was presented. Students participated in a “splitting the pills” activity where students learned how to perform simple medication calculations and fill prescriptions using a pill splitter. Accuracy of medication labeling was also emphasized during the activity.

#### Dentistry session

Students learned about good oral hygiene and career opportunities associated with a dental career (i.e. dentists, dental hygienists, dental laboratory technicians or dental assistants). A dentistry video was shown and followed by a “two-timing plaque” activity. The activity reinforced the importance of the dental profession in maintaining good oral hygiene using two-tone plaque discoloring tablets.

#### Public health session

Public health careers were explored to understand the importance of protecting the general public from infectious, chronic, and occupational health hazards. Health specialists, health educators, and epidemiologist careers were all explored during the session. A “Glo-Germ” activity was developed to understand hand hygiene and reinforce the job of public health professionals in preventing disease outbreaks.

#### Nursing session

This session was designed to bring awareness to the critical nursing shortage across the country, notably in Alabama. Nursing career options were presented and students learned about a variety of nursing degrees and specialties through a short video presentation. The “Put A Little Pressure on Me” activity was conducted by allowing students to understand what a nurse’s everyday routine involves and allowed students to practice taking blood pressure.

#### Medicine session

Students were given an overview of the requirements to complete medical training, including the admissions process, Medical College Admission Test (MCAT), and what medical school entails. The path to becoming a Physician Assistant was also discussed. A video on medicine was shown, followed by an “Operation” activity, in which students tested their hand and eye coordination with the classic game Operation, but with a twist: each time a student “buzzed” they were required to relay one fact related to becoming a physician or physician assistant to the rest of the group.

#### Nutrition/geriatric sensitivity session

Students had the opportunity to explore careers as dieticians and nutritionists, with emphasis on the importance of receiving certifications. The presentation also included a segment on breaking down stereotypes about the elderly, and an emphasis on the need for geriatric healthcare workers. A video on dieticians was shown followed by a “Taste & See” activity, during which students had the opportunity to mix and taste thickening agents used for liquids, sample pureed foods, and model healthy meals using plastic foods and calorie counting.

#### College career readiness/financial aid

This presentation was designed to provide students with practical advice and information related to post-secondary education. It covered the benefits of attending college, how to choose an undergraduate training program, and resources for paying for college, including financial aid options such as scholarships and grants. A video on college preparation and financial aid was shown and a “College and Career Readiness Edition” PowerPoint presentation was given. Additionally, students had opportunities to participate in college and university tours developed in collaboration with each participating school.

#### Dixie Health Career Never-Ending Road to Discovering Health Careers (N.E.R.D.) Summer Enrichment Program

The Dixie Health Career NERD Summer Enrichment Program was a 3-day health career exploration experience at Camp McDowell in Jasper, Alabama. The camp was designed to enrich the lives of high school students who showed a genuine interest in learning about health professions through intensive activities and interactive sessions. During the camp, students were provided with basic health skills training, educational growth, and information about resources available to them. Inclusion criteria consisted of student conduct, application fee, grade point average, past participation, and parental consent. The program consisted of a community service activity, health education service project, consent to commit to improving educational skills and developing skills to improve overall school course grades.

### Data collection

Health outcome factors in Alabama and the United States were compared to evaluate differences that exist between the state and nation (Table 1) [6, 12]. Categories include primary care providers (PCP) per 100, 000 persons, geriatric (>65 years of age) population, median household income, uninsured, Medicare beneficiaries, Medicaid beneficiaries, poverty, infant mortality, and causes of mortality. County rankings for health outcomes and factors were numbered based on severity (Table 2) [13]. Health outcomes represented the healthiness associated within each county, and were measured using how long people live and how healthy people feel while alive [13]. Health factor rankings were used to assess the health of a county and were based on measures of health behaviors, clinical care, social and economic and physical environment factors [13].

**Table 1:** 2015 Impacting health outcome factors compared between the United States and Alabama [4]

	United States	Alabama
PCP Phys/100K Pop (%)	74.5	62.8
Gen/Fam/100K Pop (%)	29.9	27.0
Internal Medicine/100K Pop (%)	29.1	23.3
Pediatricians/100K Pop (%)	58.9	47.7
OB/GYN/100K Pop (%)	21.4	18.6
Psychiatrists/100K Pop (%)	9.4	5.6
Dentist/100K Pop (%)	59.4	42.5
65+ of Total Pop (%)	13.7	14.5
Median Household Income ($)	51.371	41.610
Uninsured (%)	17.0	15.8
Total Pop Medicare Beneficiaries (%)	16.0	18.7
Total Pop Medicaid Beneficiaries (%)	20.2	19.1
Poverty (%)	15.9	19.0
Infant Mortality (per 1000 Births)	6.5	9.1
Mortality (deaths per 100, 000 population)		
All Causes	746.8	939.5
Heart Disease	179.1	236.0
Cancer	172.8	191.7
Stroke	39.1	51.6
Diabetes Related (%)	20.8	25.0
Chronic Lower Respiratory Disease (%)	42.2	55.5

**Table 2:** 2015 West Central Alabama Black Belt Health outcomes and health factors (Rank out of 67) [13]

West Central Alabama AHEC counties	Health outcomes	Health factors
Bibb	45	33
Choctaw	63	35
Dallas	53	62
Fayette	58	22
Greene	64	61
Hale	38	58
Lamar	39	44
Marengo	51	43
Perry	62	63
Pickens	41	36
Sumter	56	56
Tuscaloosa	18	12
Wilcox	65	65

Sociodemographic information from high school students was aggregated using a web-based database called iAHEC. Program characteristics necessary for successful engagement in a variety of enrichment activities were qualitatively and quantitatively evaluated for effectiveness.

The participants in the school-based enrichment program completed the pre-test and post-test via Survey Monkey to measure knowledge acquisition over the course of the program (i.e. before and after program completion); responses were gathered using the same web-based program. The Survey Monkey tutorial program assessment included 25 true and false questions designed to test current knowledge about various health care professionals. Questions included: A medical social worker creates new kinds of medicines; All doctors have to work with blood; a nursing assistant is licensed to perform surgery and prescribe medication; nutritionists help people manage chronic disease like diabetes; A phlebotomist works on people’s eyes; A nutritionist can help you develop a weight loss program; pharmacists only work in drugstores; A pharmacist is responsible for medications safety; Health administration incorporates business and management skills; Most dentists have at least 8 years of education after high school; The study of diet (what you eat) is a science; In addition to classes in physics, biology, mathematics, and chemistry, premedical students should also take English; To become a licensed physician, requires 4 years of medical school, and a minimum of 2–3 years of training after graduating from medical school in a residency program; Nurse Practitioners are registered nurses with 4 year degrees and a graduate or certificate nurse practitioners program; Public health epidemiologists work to figure out what is causing a particular disease (such as contaminated food or water); Community health workers (CHWs) work in laboratories; Some people can become certified nursing assistants while still in high school; A family physician is a doctor who is trained to take care of people of all ages, including delivering babies; A pediatrician is a medical doctor who specialized in taking care of skin; A clinical position in a hospital or clinic is one where a person works directly with patients; The only people who work in hospitals are doctors and nurses; Medical laboratory technicians help doctors figure out what is wrong with patients; Registered dental hygienist can take X-Rays; A Biostatistician collects and prepares blood for analysis.

### Data analysis

Confidence intervals were calculated at 95% to determine the response ranges for each program. The program satisfaction used a five point Likert scale which was then converted into a numerical grading system: poor = 1; below average = 2; average = 3; above average = 4; and, excellent = 5. Statistical significance was set at *P* = 0.05. One-way Anova and post-hoc tests were conducted assuming unequal variance. Graphpad Prism 6 and Microsoft Excel software were both used for analysis, tables, and figures.

## Results

### Health outcome factors

**Fig. 1:**
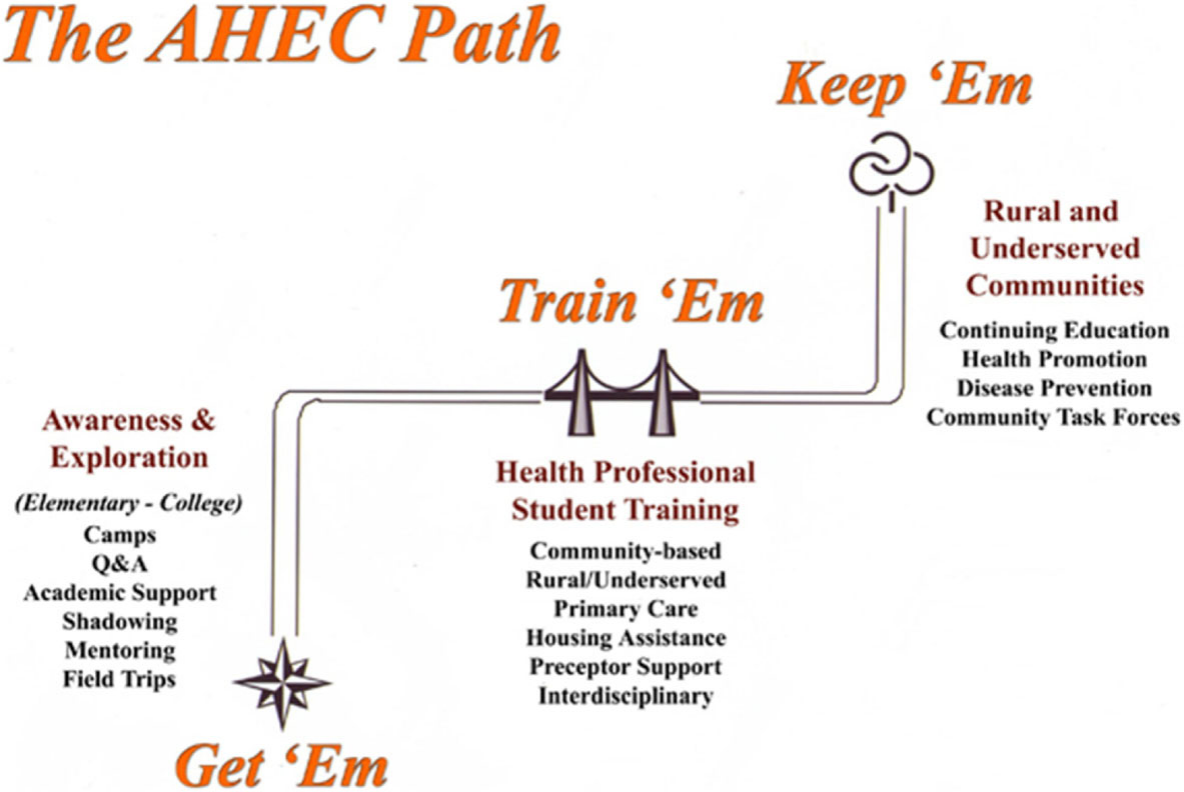
The AHEC path used to recruit and train students interested in practicing in rural and underserved communities [6]

**Fig. 2:**
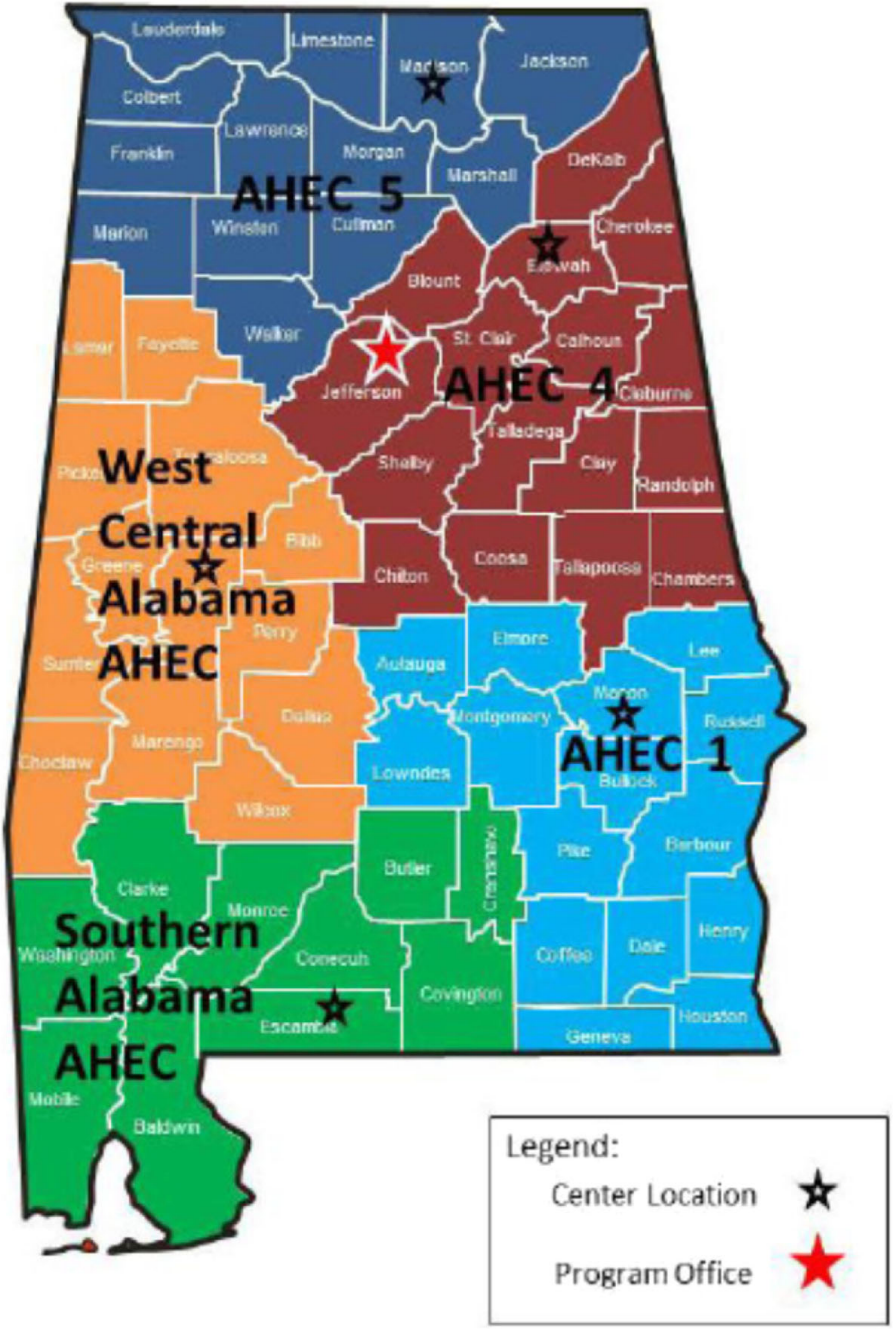
Alabama Statewide AHEC service areas [12]

**Fig. 3:**
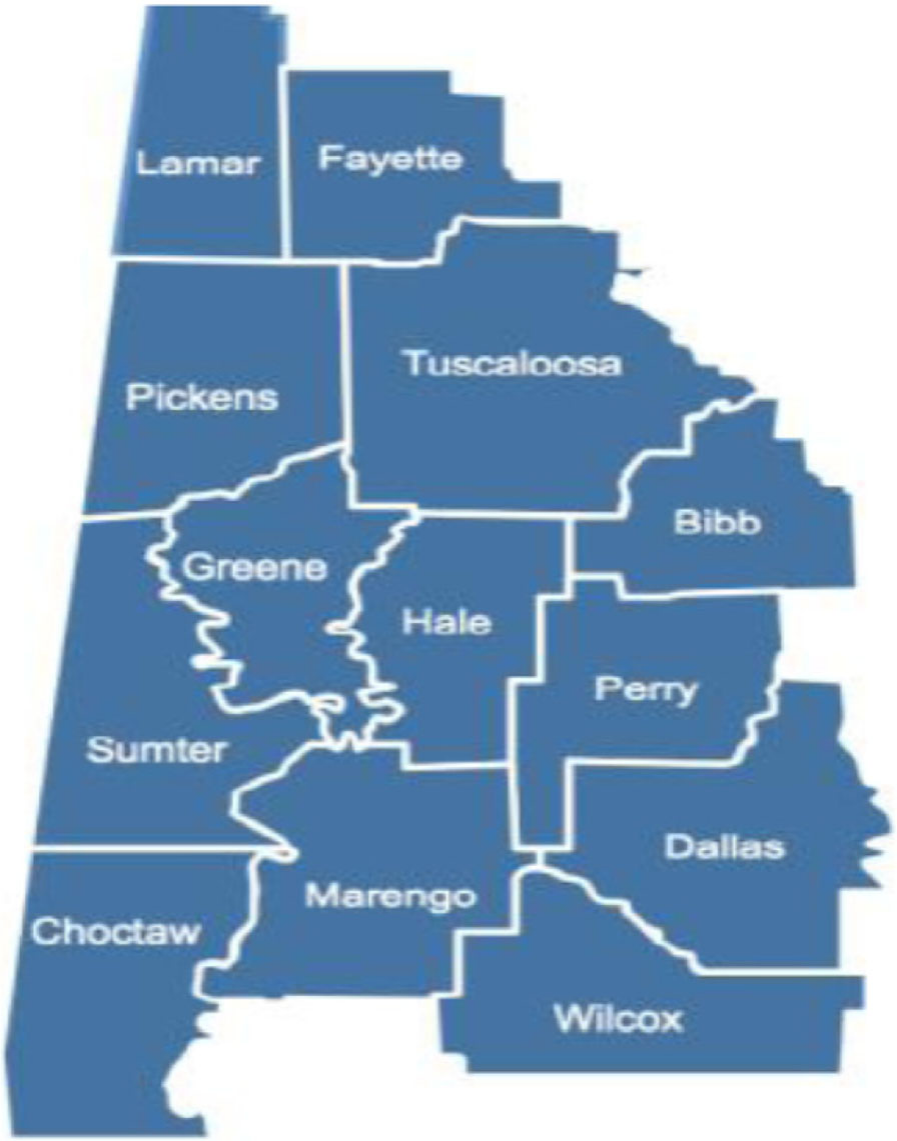
West Central Alabama AHEC service counties [12]

The development of this program followed the national AHEC logical model which places emphasis on health career awareness and exploration programs for high school students in grades 9–12. The cohort included high school students across the WCA AHEC’s 13-county service area (Figs. 1, 2,  and 3) [6, 12]. To study the importance of focusing on student recruitment in West Alabama, health outcome factors were assessed and compared to national averages (Table 1) [4]. According to these figures, Alabama has a lower prevalence of PCP per 100, 000 people and a lower average household income. In addition, there is a higher percentage of geriatric patients, uninsured individuals, Medicare beneficiaries, poverty rate, infant mortality, and adult mortality from multiple illnesses than averages found at the national level [4]. Overall, higher poverty levels and lower median incomes can significantly impact the quality of life and level of care received by each resident.

These health-related measures suggest the prevalence of poor health outcomes in Alabama when compared to the rest of the nation. It is  is crucial to plan various interventions that can help improve disease management, induce proper health behaviors, and maintain illnesses before emergency room visits are amplified and mortality rates are negatively impacted. County health rankings were categorized by number (out of 67 Alabama counties) to determine the severity of health outcomes and health factors in each of the thirteen West Central Alabama counties (Table 2) [13]. This pilot program recruited participants from the following counties: Tuscaloosa, Greene, Dallas, and Wilcox. These counties ranked in the lower half of the state for having the poorest health outcomes and health factors (Table 2) [13]. These statistics emphasize how essential it is to address poor health outcomes through tailored interventions and determine what programs could be implemented in areas to improve overall health status of individuals residing in medically underserved areas of Alabama.

### Program evaluation

Demographic data reported by each high school participant yielded a total 77.9% minority students and 61.5% disadvantaged students. A total of 593 (ACT preparation: *n* = 172, AHEC 101: *n* = 56, FAFSA: *n* = 109, Health career exploration: *n* = 159, College career readiness: *n* =67, Dixie Scholars NERD: *n* = 30) baseline program satisfaction surveys responses were assessed. Table 3 measured 95% confidence interval ranges and statistical significance for program content and presenter’s delivery ratings based on student responses.

**Table 3:** 95% confidence interval ranges for program content and presenter’s delivery

Evaluation Question/Program Title	ACT Preparation	AHEC 101	FAFSA	Health Careers Exploration	College Career Readiness	Dixie Health Career NERD
How would you rate the content of this presentation?	4.31–4.54	4.38–4.76	4.54–4.70	4.55–4.66	4.21–4.61	4.33–4.77
How would you rate the presenter's delivery?	4.32–4.56	4.37–4.71	4.57–4.73	4.59–4.78	4.22–4.60	4.06–4.56

**Fig. 4:**
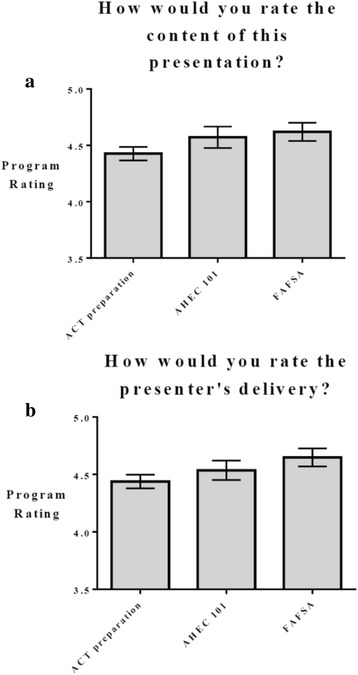
**a**) Presentation content program rating for stand-alone programs (ACT preparation, AHEC 101, FAFSA) **b)** Presenter’s delivery program rating for stand-along programs (ACT preparation, AHEC 101, FAFSA)

**Fig. 5:**
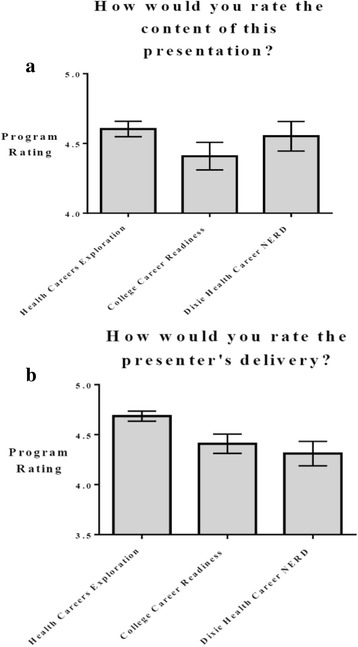
**a**) Presentation content program rating for Dixie Scholars Programs (Health Careers Exploration, College Career Readiness, Dixie Health Career NERD) **b**) Presenter’s delivery program rating for stand-along programs (Health Careers Exploration, College Career Readiness, Dixie Health Career NERD)

Participants evaluated the program by presentation content and presenter’s delivery for the stand-alone programs (ACT Preparation, AHEC 101, FAFSA) and the DASP (Health Careers Exploration, College Career Readiness, Dixie Health Career NERD). Figures  4a and b show presentation content and presenter’s delivery program ratings, respectively for each of the stand-alone programs. Figure 5a and b depict presentation content and presenter’s delivery program ratings, respectively for each of the DASP components. All programs had ratings of 4 or higher on a scale of 5 in both presentation content and presenter’s delivery. Programs were assessed for differences within each group (i.e. stand-alone and DASP), and responses were then calculated to see what differences existed between each program for content and delivery. Programs were also assessed qualitatively through student questionnaires. Statistically significant differences are reported in Tables 4 and 5 for content and delivery, respectively. Presentation content was rated different between ACT preparation and AHEC 101, Health exploration and FAFSA, with *P* values 0 .031, 0 .002 and 0 .003, respectively. Presentation delivery also had differences existing between programs. ACT preparation was found to be different in rating when compared to the Health Exploration and FAFSA programs, with *P* values of 0 .002 and 0 .033, respectively. The Dixie Health Career NERD program had different program delivery ratings with the FAFSA with *P* values of 0.041. The College career readiness and Health career exploration programs also had differences in presenter’s delivery with a P value of 0 .007.

**Table 4:** Statistically significant differences among programs - Presentation content

Program name	Program name	*P* value
ACT preparation	AHEC 101	0 .031
ACT preparation	Health Careers Exploration	0 .002
ACT preparation	FAFSA	0 .003

**Table 5:** Statistically significant differences among Programs - Presenter’s Delivery

Program name	Program name	*P* value
ACT preparation	Health Careers Exploration	0 .002
ACT preparation	FAFSA	0 .033
Dixie Health Career NERD	FAFSA	0 .041
College Career Readiness	Health Careers Exploration	0 .007

Students reported presentation content associated with ACT preparation was inferior to AHEC 101, FAFSA and, Health Career Explorations program content. The content associated with the ACT preparation program consisted of an overview of the four subject categories, test taking strategies, and a few practice examples from each category that could be implemented in a typical class setting. The FAFSA presentation allowed students to practice filling out the application online or submit their application if they had the necessary information. Health careers exploration activities included significant interactive activities, as well as a larger concentration on health career options. The ACT preparation program was not as interactive, and since the cohort of participants ranged from grades 9 through 12, some participating students may not have been college ready and in the mind-set to prepare for the standardized test.

Presenter’s delivery was also rated based on student responses. Students felt as if presentation content associated with the ACT preparation was inferior to AHEC 101, FAFSA and Health career explorations. ACT preparation was also rated lower in presenter’s delivery when compared to the FAFSA and Health career exploration programs, suggesting lower participant interest. Many students found the Dixie Health Career NERD camp to be useful, however, suggested additional interactive experiences, longer program time, and fewer classroom-associated presentations. These could be potential reasons for lower ratings when compared to the Health career explorations program, which had many interesting and interactive activities associated with a variety of health-related careers.

### Survey monkey health career pre-test and post-test

**Fig. 6:**
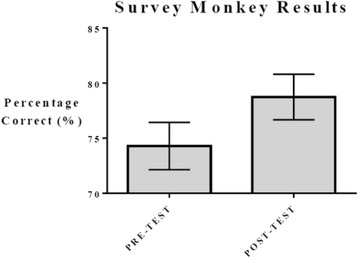
Survey Monkey results pre-test [75.18% (73.92–76.44) 95% CI] and post-test [75.15% (73.30–77.00) 95% CI]

The pre-test and post-test results included a total of 141 respondents, with a trending improvement after the completion of the program (Fig. 6). The Survey Monkey assessment showed a higher percentage of correct answers after students went through educational training; however, results were not statistically significant. Increasing the number of educational sessions that convey health related information for a longer period could show a higher change from pre-test and post-test results.

## Discussion

All five regional Alabama AHECs are non-profit organizations that receive most of their funding through a subcontract with the University of Alabama School of Medicine, that originates from the Health Resources and Services Administration (HRSA). AHECs have historically been committed to increasing the quality of health care in underserved, rural, and disadvantaged communities by providing future and current healthcare providers with appropriate training, employment opportunities and necessary resources [6]. The WCAAHEC was the first of five regional AHECs in Alabama and is still in the infrastructure development phase of programming. Most AHECs are located in geographically and medically underserved areas across the United States and are dedicated to increasing access to primary care and preventive care by enhancing the distribution of health care professionals though community and academic partnerships [7]. Various programs across the nation have been successful in increasing community-based health professions education and training opportunities that seek to recruit and retain health professionals in rural and underserved regions [7]. AHECs are designed and well positioned to be responsive to the needs of individuals that live and work in their service areas. The WCAAHEC provides services and activities that support health profession students, resident experiential education, community health awareness, and professional continuing education within the WCAAHEC 13-county service area [12].

Furthermore, the WCAAHEC serves some of the poorest counties within the state of Alabama and across the nation. In line with the mission to recruit high school students to pursue health professions in rural and underserved communities, WCAAHEC implemented the program to introduce high school students to careers in health professions by targeting schools in rural areas with large percentages of minority and disadvantaged students. These efforts were supported through a variety of activities including health career fairs, guest lectures, health presentations, shadowing, internship programs, academic development, health career education, and individualized mentoring. The pilot program participants either joined an enrichment program (DASP) or attended a stand-alone session(s) related to health professions and/or educational training.

### Grassroots healthcare workforce development

One of AHECs core programs involves recruiting students from rural and underserved communities into primary care, often referred to as health careers promotion and preparation. Students have an opportunity to participate in health career enrichment activities through health fairs, college tours, guest speakers, shadowing, health clubs, mentoring, and health careers summer camps. The WCAAHEC initial stage programs were developed to help a cohort of high school students gain an understanding of health career options and how planning is necessary to effectively matriculate into college and ultimately pursue a career as a future health care professional via additional training.

Overall, it is important to conduct program evaluations of core competencies, especially during initial stages of implementation studies, to gain a better appreciation of successful strategies for future interventions and what approaches might be more beneficial for current and future program participants. It is necessary to determine what methods are efficient in retaining students to serve similar rural and medically underserved areas. Furthermore, student perspectives provide a practical insight on what programs are superior to others, what areas need improvement, and what interventions methods are preferred for the target population or community.

### Engagement and learning: the power of interaction

Many of the programs received higher scores for presentations that were more interactive and informative for students interested in pursuing a health profession. It is important to understand the significance of interactive experiences that target current health care burdens to allow students to gain awareness of the negative impacts associated with poor health outcomes. Video usage and innovative activities that assess learned material through enjoyable testing methods provided students the ability to retain taught information, suggesting similar considerations for future initiatives. The implementation of these programs at baseline provides us the opportunity to take student suggestions and make modifications to each activity.

### Student selection

It is also important to choose students who are motivated, mature, compliant, attentive, and interested in serving AHEC counties as future health care professionals. These students can provide useful feedback about programs and learning material for better training. Students who participated in stand-alone programs like ACT preparation and College and Career Readiness may or may not have been interested in health careers. Stand-alone presentations are often presented to an entire grade without accessing their career path interest. DASP students were selected as high propensity students having an interest in health careers, and received more contact from AHEC staff on a regular basis. Furthermore, literacy and educational levels of the students can make it problematic for some individuals to obtain the most out of the sessions. 

### Recommendations

Successful implementation intervention questions to assess the effectiveness of certain programs include understanding what program content could be useful and how to tailor various aspects of the program based on student interests. Furthermore, presenters and program delivery should also be assessed from a student’s perspective to understand the effectiveness of each session, particularly from an interactive viewpoint. The act of creating an interactive environment where students and faculty are both involved should be incorporated in future retention initiatives that target minorities, rural, and disadvantaged individuals from communities located in the Alabama Black Belt. Previous research supports the use of interactive activities to foster health career aspirations for high school students from underserved communities, which will help adopt a more entertaining learning environment for prospective health professional students [14]. Future efforts to evaluate programs through participant comments, quantitative data, and qualitative information should be enhanced to obtain additional evidence to improve program implementation and assess effectiveness in meeting organizational goals. There are plans to conduct longitudinal tracking of students through post-graduation to measure the impact of implementing enrichment interventions and how significant these programs have on career choice and practice location.

## Conclusions

The baseline intervention implemented by the WCAAHEC included a large proportion of minority students residing in rural Alabama Black Belt areas that are designated as medically underserved. Student evaluations reported effective overall scores of 4′s and 5′s on a scale of 5 in presentation content and presenter delivery. Furthermore, DASP programs received higher participant feedback in comparison to stand-alone programs, potentially attributed to a larger number of interactive activities and engaged interest in health professional career topics.

## Abbreviations

ACT: American College Testing
AHEC: Area Health Education Center
DASP: Dixie AHEC Scholars Program
FAFSA: Free Application for Federal Student Aid
HPSA: Health Professional Shortage Area
HRSA: Health Resources and Service Administration
MCAT: Medical College Admission Test
WCAAHEC: West Central Alabama Area Health Education Center
